# Studying the Formation of Fullerenes During Catagenesis

**DOI:** 10.3390/molecules30122516

**Published:** 2025-06-09

**Authors:** Jens Dreschmann, Wolfgang Schrader

**Affiliations:** Max-Planck-Institut für Kohlenforschung, Kaiser-Wilhelm-Platz 1, 45470 Mülheim an der Ruhr, Germany

**Keywords:** fullerenes, ultrahigh-resolution mass spectrometry, crude oil, complex reaction mixture

## Abstract

The formation of polycyclic aromatic hydrocarbons (PAHs) during catagenesis does not exclusively lead to planar structures. The inclusion of five-ring elements increases the curvature of PAHs and yields bent molecules. These bowl-like configurations may end in the formation of spherical carbon allotropes as fullerenes or nanotubes, as recently shown. The presence of fullerenes in crude oil raises the question of why the reaction is feasible under catagenic conditions although the laboratory synthesis of fullerenes commonly requires high-energy environments. This study focuses on the feasibility of the simulation of catagenesis under laboratory conditions and the question of which building blocks may lead to spherical structures. Possible educts, reaction mechanisms, and conditions such as temperature are discussed and related to experimental outcomes. For the simulation under laboratory conditions, a light gas condensate was fractionated by distillation in order to reduce the number of compounds per fraction and make them distinguishable. The characterization of the resulting fractions was performed through GC-MS and GC-FID measurements before heat application in a closed reactor. High-resolution mass spectrometry (HRMS) measurements of the products indicated PAH growth and, more importantly, the formation of fullerenes. Interestingly, the characterized fullerenes mostly comprised the range of non-IPR (isolated pentagon rule) fullerenes.

## 1. Introduction

**Figure 1:**
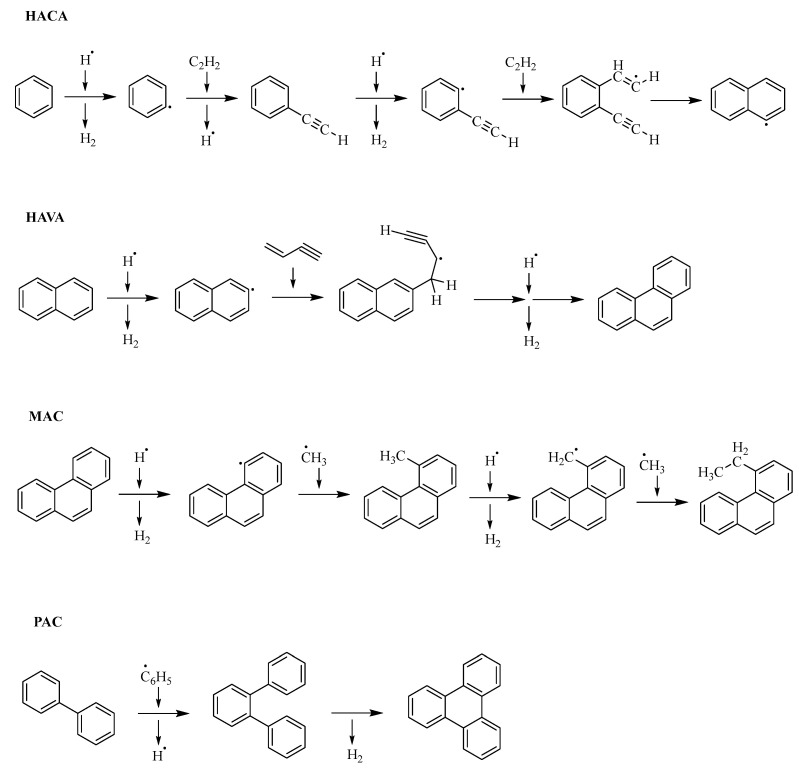
Summary of different reaction mechanisms [11].

The formation of crude oil—widely known as catagenesis—is a process that includes the slow carbonization of dead organic matter [1]. The products comprise hydrocarbons such as alkanes, alkenes, and naphthenic or aromatic compounds, but depending on the elemental composition, the product spectrum also contains heteroatom (N, O, S)-containing compounds. Large aromatic compounds, either with or without heteroatoms, can be contained in the fraction called asphaltenes [2], which makes up a large portion of heavier crude oil and causes many adverse effects during refinement [3,4]. The growth process of aromatic or polycyclic aromatic hydrocarbons (PAHs) obviously starts with the first aromatic ring and theoretically continues indefinitely. The chemical feedstock for the initial aromatic ring fusion originates from cracking under natural circumstances, producing light alkanes as well as alkenes [5]. Light alkanes and alkenes then may form the first aromatic ring by radical or carbocation reactions, which can induce subsequent hydrogen elimination [6,7,8,9,10]. Manifold reaction pathways may lead to the formation of aromatic species. 1,3-butadiene and acetylene make up the bulk of building blocks in these reactions [11]. The growth mechanisms of PAHs can be categorized by their respective reactants, and some of them are summarized in Figure 1 [11]. The hydrogen abstraction acetylene addition (HACA) mechanism and the Diels–Alder reaction both rely on the presence of acetylene. Diels–Alder is generally slower than HACA and, thus, probably plays a subordinate role [12,13,14]. Different reaction mechanisms are discussed for the formation of symmetrical PAHs like coronene or corannulene. They can be formed either through HACA or in combination with methyl addition cyclization (MAC) and phenyl addition cyclization (PAC) mechanisms [11,15]. MAC and PAC belong to the second category of mechanisms, which utilizes the most diverse group of reactants and is determined by radical additions [11]. The last reaction type leading to the growth of PAHs is called the hydrogen abstraction vinylacetylene addition (HAVA) mechanism and is based on vinylacetylene as a precursor [11]. In general, radical cyclization mechanisms tend to be more efficient and faster than HACA or HAVA [15,16,17].

The insertion of five-membered rings disrupts the planar structure of PAHs and adds curvature. Thus, the addition of pentagons may lead to hemispherical or spherical structures, such as recently discovered fullerenes or nanotubes in crude oil demonstrate [18,19]. The discovery of fullerenes in crude oil is remarkable since their formation is commonly observed during high-energy events such as arc discharges or lightning strikes, while crude oil forms under relatively mild conditions [20,21]. The simulation of this geologic process in the laboratory may provide information that helps to understand the formation mechanisms of these spherical compounds. In previous studies on crude oil fouling, the formation of fullerenes was observed [22,23,24]. These studies were based on a gas condensate—a very light crude oil fraction—and were conducted in a closed environment at elevated temperatures.

Gas condensates contain only relatively light hydrocarbon compounds, including alkanes, alkenes, and small aromatics, albeit over a broad compound range. Thus, a gas condensate represents an excellent starting material to study PAH growth under geology-near conditions. On the one hand, the compound diversity enables different PAH growth mechanisms to take place concurrently due to the presence of the different required educts. On the other hand, the diversity increases the complexity, which complicates possible inferences after the reaction. Thus, fractionation and characterization of the initial mixture are essential to counter the complexity and enable the drawing of conclusions. As the constituents of a gas condensate are primarily volatile, fractionated distillation followed by GC-FID and GC-MS analyses is a suitable choice for sample simplification and characterization [25,26].

However, when reacting a gas condensate, compounds of higher molecular weight are formed that are no longer volatile and amenable to gas chromatographic separation [22]. The expected products span a mass and complexity range that closely resembles that of crude oil [27,28]. For the analysis of crude oil and related samples, high-resolution mass spectrometry (HRMS), employing atmospheric pressure ionization (API) techniques like electrospray (ESI), atmospheric pressure chemical ionization (APCI) and atmospheric pressure photoionization (APPI), has proven to be the method of choice [11,29,30,31,32,33,34,35,36,37,38,39,40,41]. Here, we study the formation of fullerenes from a light gas condensate to help understand their formation process and mimic their geological formation in heavy crude oil. Detailed data were obtained using ultrahigh resolution mass spectrometry.

## 2. Results and Discussion

The natural formation of crude oil occurs under very diverse conditions in terms of temperature and pressure gradients as well as oxygen content. In summary, the reaction conditions vary significantly in an oil reservoir over long periods [42]. Additionally, organic matter, as the reaction feedstock, undergoes highly complex transformation processes with overwhelming numbers of reaction pathways [43]. This complexity makes it impossible to understand single reaction pathways, as the process contains a vast array of competing reactions. Other important factors beside the educt material might be the amount of available oxygen, which certainly is limited and chemically bound within the natural organic matter, as well as the influence of the source rock itself as a potential catalyst for many reactions [44,45]. The simulation of these natural conditions under laboratory conditions can only be met partially and under limited experimental conditions. In the end, the formation of crude oil takes place in a closed reaction vessel with a limited amount of oxygen. As a first approximation, a simple setup was used in this study to understand the basics of fullerene formation in crude oil. During synthesis, fullerenes are formed under very high temperatures, usually from soot. Under natural geological conditions, crude oil with all its compounds is formed at temperatures not exceeding 150 °C with high pressure but over a long time frame [46,47]. To reduce the reaction times, here the temperature was increased up to 450 °C. The starting material, a gas condensate, contained only small hydrocarbon compounds but still had a high complexity and probably contained several thousands of different compounds, including isomeric variants. For simplification and reduction of the number and types of compounds present during the reaction, the gas condensate was distilled into different distillation cuts.

### 2.1. Characterization of the Starting Material

**Figure 2:**
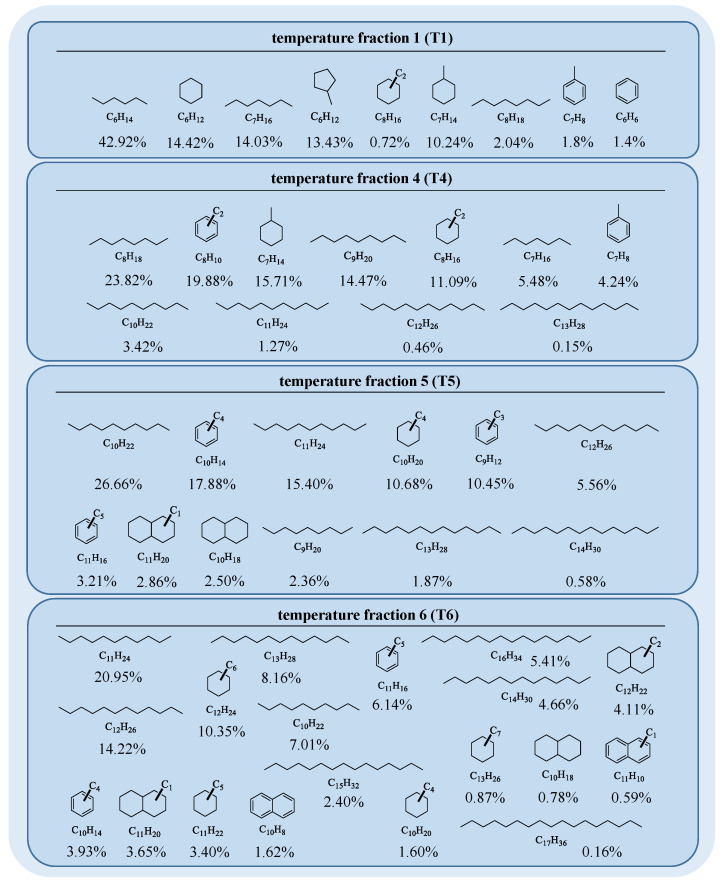
The shown structures match with the elemental compositions assigned from GC-MS data. The percentages below correlate to the peak areas of the GC-FID measurement normalized to one carbon and only include compounds with assigned elemental composition (for more details see the Supplementary Materials). Linear and branched alkanes are depicted as the respective *n*-alkane and their peak areas are summarized.

The characterization and structural elucidation of the gas condensate with GC-MS revealed alkanes and alicyclic and aromatic hydrocarbons in the original gas condensate, with molecular masses from 86 Da (hexane) up to a maximum of 240 Da for heptadecane (Figure 2); however, octa- and nonadecane were also found in traces. Following, the starting material was characterized using GC/MS to document the starting material. It has to be noted though that some detected signals were not identified and therefore omitted from the data in Table 1. Temperature fraction T2 showed similarities to T1, while T3 was more similar to T4 and, therefore, these two fractions were not further considered for this study (for more details see Figure S1 in the Supplementary Materials). The GC-FID measurements indicated hydrocarbons with carbon numbers from C_6_ to C_8_ to be most dominant in T1 (Figure 2). These hydrocarbons comprised linear and branched alkanes (≈59%) and cycloalkanes (≈39%), as shown in Table 1. Aromatic components such as benzene and toluene also appeared with smaller peak areas (≈2%). The calculated percentages included only compounds identified through GC-MS, which means that the total measured peak area in GC-FID was actually higher than the assigned peak area. In the case of temperature fraction T1, the percentage of unassigned peak area made up 12.86% (see Table S1 in the Supplementary Materials). This unassigned range mostly indicated traces of alkane isomers but also higher alkylated cycloalkanes and aromatic hydrocarbons with up to three carbon atoms in the alkyl chain. The hydrocarbons in temperature cut T4 ranged from C_7_ to C_13_ and comprised alkanes (≈49%), higher substituted cycloalkanes (≈27%), and aromatic compounds (≈24%), as can be seen in Figure 2 and Table 1. The percentage of unassigned peak area made up 33.63% and, likewise as in T1, the GC-MS spectra indicated traces of alkane isomers, alkylated cycloalkanes, and aromatics with up to five carbon atoms in side chains (see Table S2 in the Supplementary Materials). In contrast to T1, the amount of alkanes and cycloalkanes decreased while the relative amount of aromatic compounds drastically increased.

This trend roughly continued for T5 in the carbon number range from C_9_ to C_14_. Here, cycloalkanes (≈16%) numerically further decreased while alkanes (≈52%) slightly increased. The aromatic fraction (≈32%) showed the highest percentage of all fractions as well as the highest percentage of unassigned peak area (42.67%). The unassigned peak area again showed mixed fragments of alkane isomers, alkylated cycloalkanes, and aromatics, with up to nine carbon atoms in the alkyl chains (see Table S3 in the Supplementary Materials). Furthermore, traces of naphthalene and alkylated derivatives could be found. Compounds in T6 ranged from C_10_ to C_17_ and corresponded to alkanes (≈64%), cycloalkanes (≈25%), and aromatic hydrocarbons (≈11%). The amount of alkanes marked the highest count of all fractions, while the aromatic fraction was relatively low. The unassigned peak area made up 33.13% and contained alkane isomers, alkylated cycloalkanes, and aromatics (see Table S4 in the Supplementary Materials). The alkyl chains indicated lengths of up to 13 carbon atoms. The alkane content in the temperature fractions was exclusively the highest portion and varied between 49% and 64% in the fractions. Larger amounts of aromatics were found in T4 and T5, while the amounts of aromatics in T1 and T5 were significantly smaller. The amount of cycloalkanes continuously decreased from T1 to T5 and increased again in T6. The biggest aromatic core structure present in the total gas condensate was naphthalene substituted with alkyl chains of up to four carbon atoms. The degree of alkylation on aromatics and cycloalkanes increased from T1 to T6. In the end, the separation led to temperature fractions with distinguishable contents of saturated and unsaturated hydrocarbons. This differentiation allowed at least inferences on reactions of aromatic, naphthenic, and paraffinic hydrocarbons with regard to the formation and growth of PAHs.

### 2.2. Temperature Dependency of PAH Growth

The gas condensate and the distilled temperature fractions were reacted at 350 °C and 450 °C. The obtained products were investigated with a focus on PAH growth. The applied reaction temperatures clearly led to different outcomes for the products. The emerging mixture of solid-black and viscous-brown product at 450 °C pointed to coke formation. The non-soluble solid product was investigated previously and contained mostly (95 wt.%) amorphous carbon [24]. Overall, the mass balances of the reactions at 450 °C showed mass losses from 81% up to 93% (see Figure S2 in the Supplementary Materials for details). The mass losses most likely arose from thermal cracking reactions of linear or branched hydrocarbons in the starting material causing the formation of small alkanes, alkenes, alkynes, or CO_2_. The high vapor pressure of these compounds probably caused evaporation during the opening of the reaction vessel, although the metal autoclaves were cooled down to ambient temperature. Furthermore, the mass losses among the different fractions indicated no clear correlation with the differing paraffinic, naphthenic, or aromatic hydrocarbon content. The cracking efficiency should roughly increase from aromatic hydrocarbons to naphthenic hydrocarbons, and paraffinic hydrocarbons should indicate the highest efficiencies [48]. The alkylation of aromatic compounds also influences the efficiency—the longer the chain, the lower the efficiency. However, thermal cracking of alkylated aromatics yields the respective olefin and the bare aromatic molecule, and thus only partially contributes to mass loss. In general, correlations between the mass losses and the different hydrocarbon classes within the gas condensate were difficult since the portion of unassigned and unidentified compounds in the temperature fractions was significantly high, with up to 43%. Nevertheless, the mass losses indicated high cracking efficiencies, which seemed unlikely for high contents of naphthenic or aromatic hydrocarbons [49]. Thus, the actual percentage of naphthenic and aromatic compounds in the fractions was most likely lower and the unidentified part contained more alkane isomers. Another indicator for cracking reactions beside the mass losses at 450 °C was the reactions of the original gas condensate at 350 °C. GC-MS and GC-FID studies of the reaction products after reaction at 350 °C indicated reduced peak areas of bigger alkanes from *n*-nonane to *n*-hexadecane in comparison to the unreacted gas condensate, while the peak areas of *n*-heptane and *n*-octane increased (for more details see Figure S3 in the Supplementary Materials). Smaller alkanes could not be detected, probably due to the aforementioned evaporation losses while opening the reaction vessels.

**Figure 3:**
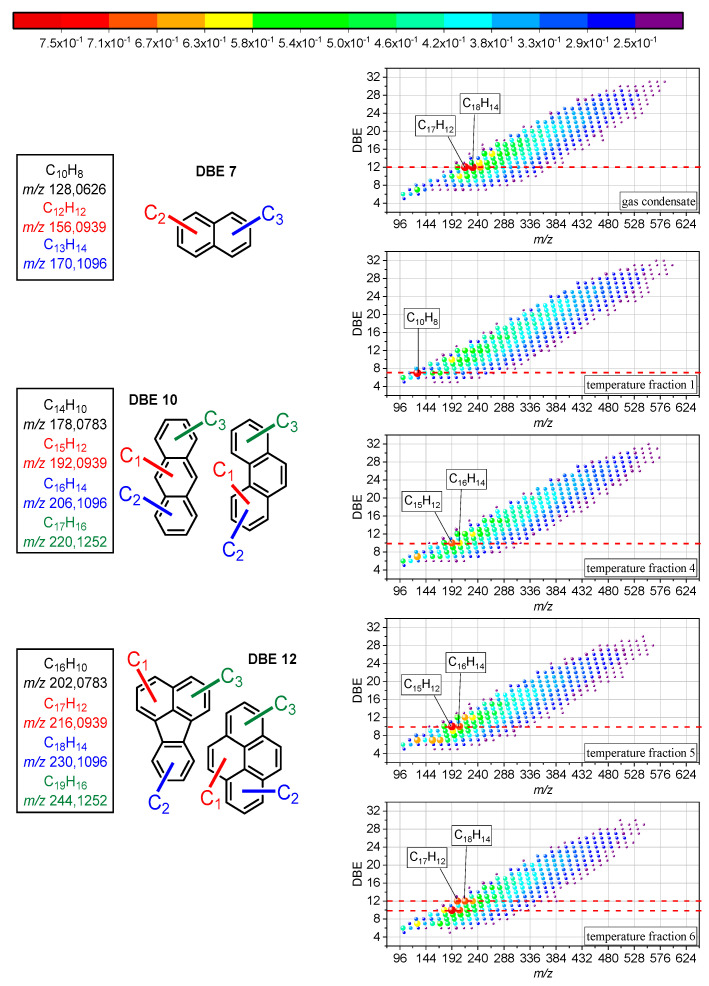
DBE vs. *m/z* diagrams of hydrocarbons detected as radical cations after reaction of the entire gas condensate and individual temperature cuts at 450 °C. The color and bubble size code the relative signal intensities of the constituents. On the left side, the proposed structures of the compounds with highest signal intensity are shown.

The formation of coke is usually an undesirable side effect during thermal cracking, but in this case, it indicated the formation of carbonaceous material, and the extracted liquid product at 450 °C showed an increase in molar mass and aromaticity, indicating the growth of PAHs (Figure 3). The detected hydrocarbon radical ions from the reacted gas condensate and its distillation fractions under air were plotted by their *m*/*z* against the corresponding double bond equivalent (DBE), indicating signals from around 100 to 600 Da and a DBE of 5 to 32. The span of the distribution slightly diminished from T1 to T6 by around 50 Da, which pointed to certain growth reactions of PAHs. Smaller alkanes require less time to yield the final cracking product since fewer consecutive reactions are involved [50]. Ethene probably made up a considerable amount of the cracking product yield and likely fueled PAH growth as precursor of the HACA route [49]. Acetylene, as the main reactant of the HACA route, can also form under thermal cracking conditions, albeit in minor amounts (k_(900 K)_ = 4.9 × 10^−3^ s^−1^) [50]. The unlikely formation of vinyl acetylene during thermal cracking ruled out HAVA as a substantial formation route of PAHs. In the end, the reaction kinetics impeded PAH growth due to the precursor formation slowing down with increasing chain length of the alkanes since increasing the chain length involves more consecutive reactions. Long residence times, as in this case, enable the formation of methane or methyl radicals beforehand, which may react as educts in the MAC mechanism to promote PAH growth [51,52]. The MAC mechanism, as a competing reaction with HACA, is faster and more efficient for growing PAHs, and methyl radicals are probably more available than acetylene since methane is formed in multiple cracking reactions (k_(900 K)_ = 4.94 × 10^5^ s^−1^ highest reaction rate from cracking pentene) [50]. In the end, both reactions may have taken place and contributed to the growth of PAHs during the reaction.

The formation of large PAHs requires aromatic precursors and, in case of the first temperature fraction, only small amounts of aromatics were present. Nevertheless, the distribution in Figure 3 indicated the highest molecular mass and aromaticity increase for T1. Thus, the formation of the first aromatic ring seemed to pose no hindrance with regard to the reaction kinetics. The most likely routes here were probably the reaction of either a butadienyl radical with acetylene or butadiene with a vinyl radical [11]. The butadiene yielded from the thermal cracking of hydrocarbons should be sufficient as a cracking product of pentene (k_(900 K)_ = 4.94 × 10^5^ s^−1^) [50,53,54,55]. The amount of vinyl radicals yielded from multiple reactions from ethene (k_(900 K)_ = 6.59 × 10^6^ s^−1^ highest for cracking of *n*-heptane) is probably higher in comparison to the amount of acetylene yielded from propene cracking (k_(900 K)_ = 4.9 × 10^−3^ s^−1^) [50]. This made the reaction of ethene and butadiene more likely (k_(900 K)_ = 2.78 × 10^1^ s^−1^) [50]. The overall impression of the product distributions in Figure 3 suggested similar results. However, a closer look at the relative intensities indicated differences in the distribution of the intensity maxima. The most abundant signals for the reaction of the entire gas condensate were at a DBE of 12. The DBE of the highest signals of the distillation fractions increased from 7 in T1 to over 10 in T4 and T5 and to 12 in T6. The fragmentation of the compounds that corresponded to the highest signal with a DBE of 7 indicated naphthalene and derivatives (see Figures S4–S6 in the SI). Compounds at a DBE of 10 revealed aromatic core structures of anthracene or phenanthrene (see Figures S7 and S8 in the Supplementary Materials). The formation of phenanthrene is thermodynamically favored in comparison to anthracene [56]. The fragmentation pattern of the compounds that corresponded to a DBE of 12 matched with pericondensed PAHs such as fluoranthene or pyrene and alkylated derivatives (see Figures S9–S12 in the Supplementary Materials).

The DBE increase might have been related to another compound group in the temperature fractions. The aromatic hydrocarbon content may explain the described increase. T1 only contained limited amounts of benzene and toluene and probably formed aromatic rings from cracking products. The highest signals indicated naphthalene as the most prominent PAHs. The portion of aromatic hydrocarbons in T4 and T5 significantly increased and thus indicated most likely phenanthrene and alkylated derivatives as the most dominant PAHs. In T6, the most prominent PAHs were either alkylated fluoranthene or pyrene, although the aromatic content was smaller in comparison to T4 and T5. However, T6 contained naphthalene and alkylated derivatives, which already exhibited a DBE of 7. The size of the already present precursors increased, while at the same time, the DBE of the highest intensity compounds increased. The involved reaction mechanism pointed to HACA or MAC since PAC would yield a biphenyl intermediate (C_12_H_10_), which was not observed at elevated signal intensity [11]. This would also match with the overall observation of successive PAH growth. Compounds such as phenanthrene or fluoranthene may have acted as intermediates or precursors on the way to building fullerenes.

### 2.3. Formation of Fullerenes During Reaction

**Figure 4:**
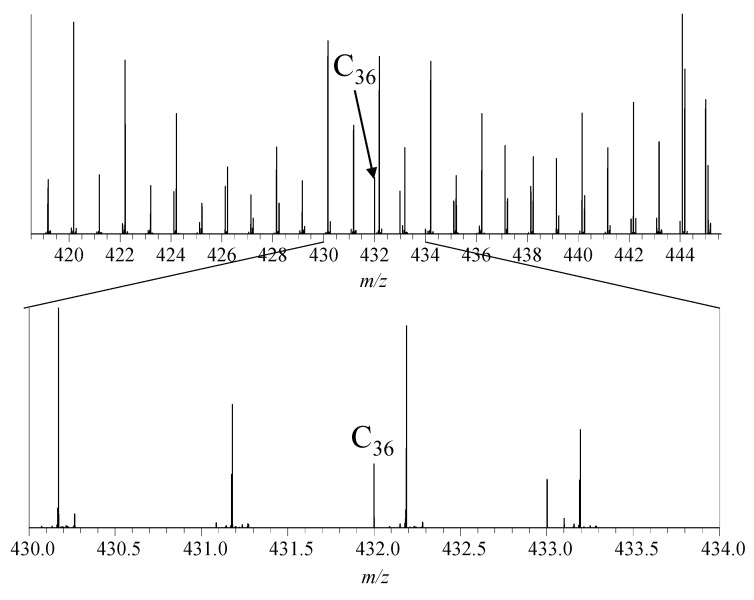
Mass spectrum of the gas condensate after reaction at 450 °C, zoomed in region around *m*/*z* 432, showing the signal of C_36_^+^.

Overall, the mass spectrometric investigations of all reactions indicated the formation of fullerenes (see Figure 4).

**Figure 5:**
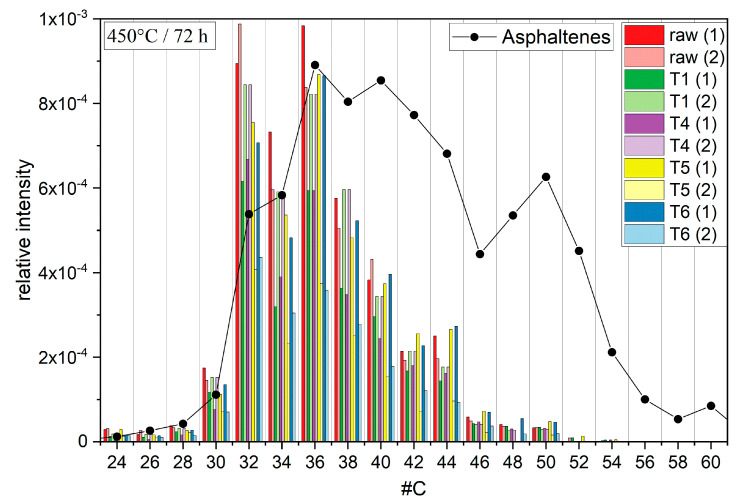
Bar chart diagram of fullerene signals in the reaction products of the initial gas condensate (red), T1 (green), T4 (purple), T5 (yellow), and T6 (blue). The height of the bars correlates with the signal intensity relative to the tallest peak. The black dotted line represents the natural fullerene distribution in asphaltenes.

Surprisingly, the formation of non-IPR (isolated pentagon rule) fullerenes seemed to dominate the reaction since the signal intensities of IPR fullerenes such as C_60_ were present only in minor amounts (Figure 5). Non-IPR fullerenes have structures with connected 5-membered ring systems, while in IPR fullerenes, the 5-membered rings are surrounded by 6-membered rings only. The comparability of the signal strength and the abundance of fullerenes in the mass spectrometric measurements were discussed in a previous study [57].

These results raise the question of why non-IPR fullerenes are preferred as reaction products compared to more stable IPR fullerenes. The formation of carbon nanotubes (CNTs) or fullerenes from carbonaceous material under thermal conditions is widely accepted [58,59,60,61]. The type of carbon source with the ability to decompose into sp^2^ or sp carbon species is essential for growth, as is the presence of catalysts [62].

The results presented here show the formation of fullerenes without catalysts and at lower temperatures, albeit the signal intensities indicated only small yields. The signal intensities of the observed fullerenes varied strongly, probably due to their low abundance (Figure 5). The distributions of the different fractions indicated no clear trend of specific fullerenes being preferably formed (Figure 5). These results matched with previous observations and regular PAH growth through HACA and MAC mechanisms. These mechanisms would at least explain the randomly distributed intensities when comparing the different temperature fractions. The absence of bigger fullerenes such as C_60_ might be explained by the presence of oxygen. Previous publications indicated a correlation between the ratio of molecular and atomic oxygen and the growth tendency of PAHs [63]. A high ratio of molecular over atomic oxygen promotes the growth of smaller PAHs during pyrolysis. The formation of atomic oxygen was probably impeded in the reaction setup due to the relatively low temperatures, which explains the relatively small molecular masses found in the PAH distributions (Figure 3). These PAH distributions in turn may explain the fullerene distribution, since the highest molecular masses of the PAHs barely reached 600 Da. The fullerene distribution likewise indicated only small abundances of fullerenes bigger than C_50_ (600 Da). Thus, fullerene growth may also have been influenced by the ratio of molecular over atomic oxygen, and higher molar fractions of molecular oxygen may lead to the formation of smaller fullerenes. In the end, the absence of molecular oxygen during catagenesis may explain the formation of non-IPR fullerenes in heavy crude oil fractions.

The distribution of fullerenes in asphaltenes showed a significant shift in comparison to the distributions of the temperature fractions in this reaction setup. The fullerene distribution in asphaltenes showed much higher signals, with C_148_ found as the largest fullerene, and maximum shifts to higher molecular masses and carbon numbers of up to 60, while the fullerene distribution of the reaction mixture from the temperature fractions indicated maxima at 32 and 36 carbon atoms and a steady decrease in intensity with increasing carbon number. On the one hand, the formation of fullerenes in crude oil under natural conditions may involve reaction mechanisms other than HACA or MAC, which could not be emulated under the conditions used in this work. On the other hand, non considered factors such as catalysts, pressure, other precursor molecules, or simply longer reaction times may contribute to the formation process of fullerenes and CNTs in crude oil.

## 3. Conclusions

The distillation of this gas condensate provided fractions with unique characteristics that enabled the collection of basic information about the involved educts. Heat application on the gas condensate and the distillation fractions indicated PAH growth through the HACA and MAC mechanisms. The formation of fullerenes in the range from C_20_ to C_50_ was observed in all temperature fractions as well as in the original gas condensate. No clear fullerene formation tendencies were observed with regard to the different temperature fractions and their different portions of alkanes and naphthenic or aromatic compounds. Thus, fullerene growth probably also involved mechanisms such as HACA or MAC. The maximum size of fullerenes matched with the highest molar masses of the formed PAHs, which might be related to the ratio of molecular over atomic oxygen available during the process. In conclusion, the fullerene growth in the experiments might have been influenced by a higher molar fraction of molecular oxygen. The fullerene distribution of the products significantly varied from the fullerene distribution in asphaltenes. However, the natural conditions of crude oil catagenesis also differ significantly in comparison to the experimental approach and the inclusion of factors such as catalysts or other precursors may lead to another product distribution in future experiments. Nevertheless, the experiment successfully demonstrated the formation of fullerenes from a light hydrocarbon mixture under relatively low temperatures in comparison to other pyrolysis approaches that aim to synthesize fullerenes.

## 4. Materials and Methods

Sample Preparation. A light gas condensate from a natural gas reservoir was utilized for all experiments. The gas condensate was distilled into six temperature fractions (T1–T6), with T5 and T6 distilled at a reduced pressure of 30 mbar. The distillation temperatures were measured according to the oil bath temperature: T1: 20–120 °C (1 atm), T2: 120–140 °C (1 atm), T3: 140–160 °C (1 atm), T4: 160–180 °C (1 atm), T5: 20–100 °C (30 mbar), T6: 100–150 °C (30 mbar). The original sample and all temperature cuts were diluted in dichloromethane to a final concentration of 1000 µg/mL for GC analysis. Reacted samples were diluted in toluene and methanol (50:50, *v/v*) to a final concentration of 300 µg/mL for direct infusion HRMS.

**Figure 6:**
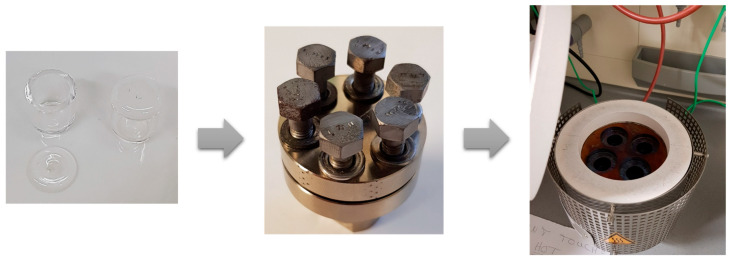
Experimental setup. The left picture shows the quartz reaction crucibles, the middle picture shows the airtight autoclave, and in the right picture, the heating device is shown.

Simulation of crude oil genesis. Stainless steel autoclaves serve as modeling vessels for the reaction. Quartz crucibles functioned as sample containers and were used as inserts for the autoclaves. A loosely placed quartz lid with a small opening for gas exchange covered the crucibles. The autoclaves were closed with stainless steel seals to enable the reaction to occur under different gaseous environments (Figure 6). The experimental setup was adapted from former experiments [22,23,24]. This study included the reaction under an ambient air atmosphere. Experiment temperatures were varied up to a maximum of 500 °C, which was controlled by a laboratory-built heating device, and the reaction time was set to 72 h. The reacted samples were extracted with anisole, ultrasonicated, and filtered through filter paper (Whatman, Buckinghamshire, UK) to retain the solid material. The filtered liquid was evaporated to complete dryness under a gentle nitrogen stream. For analysis, the sample material was reconstituted in a mixture of anisole and heptane (50:50, *v/v*) and diluted to a final concentration of 300 µg/mL.

Instruments and methods. The untreated gas condensate as well as the distilled fractions were characterized using a Q Exactive GC system (Thermo Fisher Scientific, Bremen, Germany) over a MS scan range of *m*/*z* 30–750 at a mass resolving power of *R* = 120,000 (FWHM at *m*/*z* 200). The electron ionization (EI) source was operated at 250 °C using an electron energy of 70 eV after an initial delay time of 30 s. Gas chromatography was performed on a Rtx-1 capillary column (30 m × 0.25 mm id × 0.25 µm film thickness, with 7 m pre-column, Restek GmbH, Bad Homburg, Germany) at a constant helium (N5.0) carrier gas flow of 1.2 mL min^−1^. The temperature gradient was a 10 °C min^−1^ ramp from 35 °C to 280 °C. The temperature transfer line and injection port were set to 250 °C. Split mode was engaged with a split flow of 90 mL min^−1^, except for fraction T3 with a split flow of 30 mL min^−1^. The injection volume was 1 µL. This method was adopted for the GC-FID measurements; however, the used DB-1 capillary column differed slightly (30 m × 0.25 mm id × 0.25 µm film thickness, without a pre-column, Agilent Technologies, Germany). The GC-FID measurements were performed using an Agilent GC 6890 N GC-FID system (Agilent Technologies, Waldbronn, Germany).

Other measurements were performed using a research-type Orbitrap Elite mass spectrometer (Thermo Fisher Scientific, Bremen, Germany) with a mass resolving power of *R* = 480,000 (FWHM at *m/z* 400). Positive polarity spectra were acquired in spectral stitching mode using windows of 27 Da with a 3 Da overlap. For each window, 7 microscans were added to provide adequate data depth. Ionization by APCI was accomplished with a corona discharge needle to generate a cold plasma at a current of 4.5 µA. The heated sprayer was operated at 350 °C using nitrogen as the sheath gas in both cases.

Data analysis. The acquired data were analyzed using Xcalibur software (Thermo Fisher Scientific, Bremen, Germany) and Composer V1.5.0 software (Sierra Analytics, Modesto, CA, USA) for elemental formula assignment. For peak assignments, the following criteria regarding the number of possible elements and the number of double bond equivalents (DBE) were applied: 0 < H < 1000, 0 < C < 200 and 0 < DBE < 120, with a maximum mass error of 1 ppm.

## Figures and Tables

**Table 1:** Proportion of different hydrocarbon classes in the distillation fractions of the gas condensate (for more details see Figures S2–S5 in the Supplementary Materials).

Fractions	Hydrocarbon Classes (Relative Amounts of the Total Assigned Peaks [%])		
	Alkanes	Cycloalkanes	Aromatics
T1	58.99	38.80	2.21
T4	49.08	26.80	24.12
T5	52.42	16.03	31.54
T6	64.01	25.17	10.83

## Data Availability

Data are documented within the article and the Supporting Information.

## Supplementary Materials

The following supporting information can be downloaded at: https://www.mdpi.com/article/10.3390/molecules30122516/s1, Figure S1: Gas chromatograms (GC-MS) of the initial gas condensate; Table S1: Summary of compounds in temperature fraction 1; Table S2: Summary of compounds in temperature fraction 4; Table S3: Summary of compounds in temperature fraction 5; Table S4: Summary of compounds in temperature fraction 6; Figure S2: Mass balances of the reaction of the gas condensate, T1, T4, T5, and T6; Figure S3: Peak areas from GC-FID measurements; Figure S4: Fragmentation spectrum of C_10_H_8_; Figure S5: Fragmentation spectrum of C_12_H_12_; Figure S6: Fragmentation spectrum of C_13_H_14_; Figure S7: Fragmentation spectrum of C_15_H_12_; Figure S8: Fragmentation spectrum of C_16_H_14_; Figure S9: Fragmentation spectrum of C_16_H_10_; Figure S10: Fragmentation spectrum of C_17_H_12_; Figure S11: Fragmentation spectrum of C_18_H_14_; Figure S12: Fragmentation spectrum of C_19_H_16_. References [64,65,66] are cited in the supplementary materials.
