# Supplementary material for: Studying the Formation of Fullerenes During Catagenesis
Source: Molecules. 2025 Jun 9;30(12):2516. doi: 10.3390/molecules30122516 (PMC12196268; doi:10.3390/molecules30122516)
Supplement: Supplementary file 1 [file molecules-30-02516-s001.zip › molecules-3630625-supplementary.pdf]

Supplementary Materials

Studying the Formation of Fullerenes During Catagenesis

Jens Dreschmann and Wolfgang Schrader *

Max-Planck-Institut für Kohlenforschung, Kaiser-Wilhelm-Platz 1, 45470 Mülheim an der Ruhr, Germany;
* Correspondence: wschrader@kofo.mpg.de

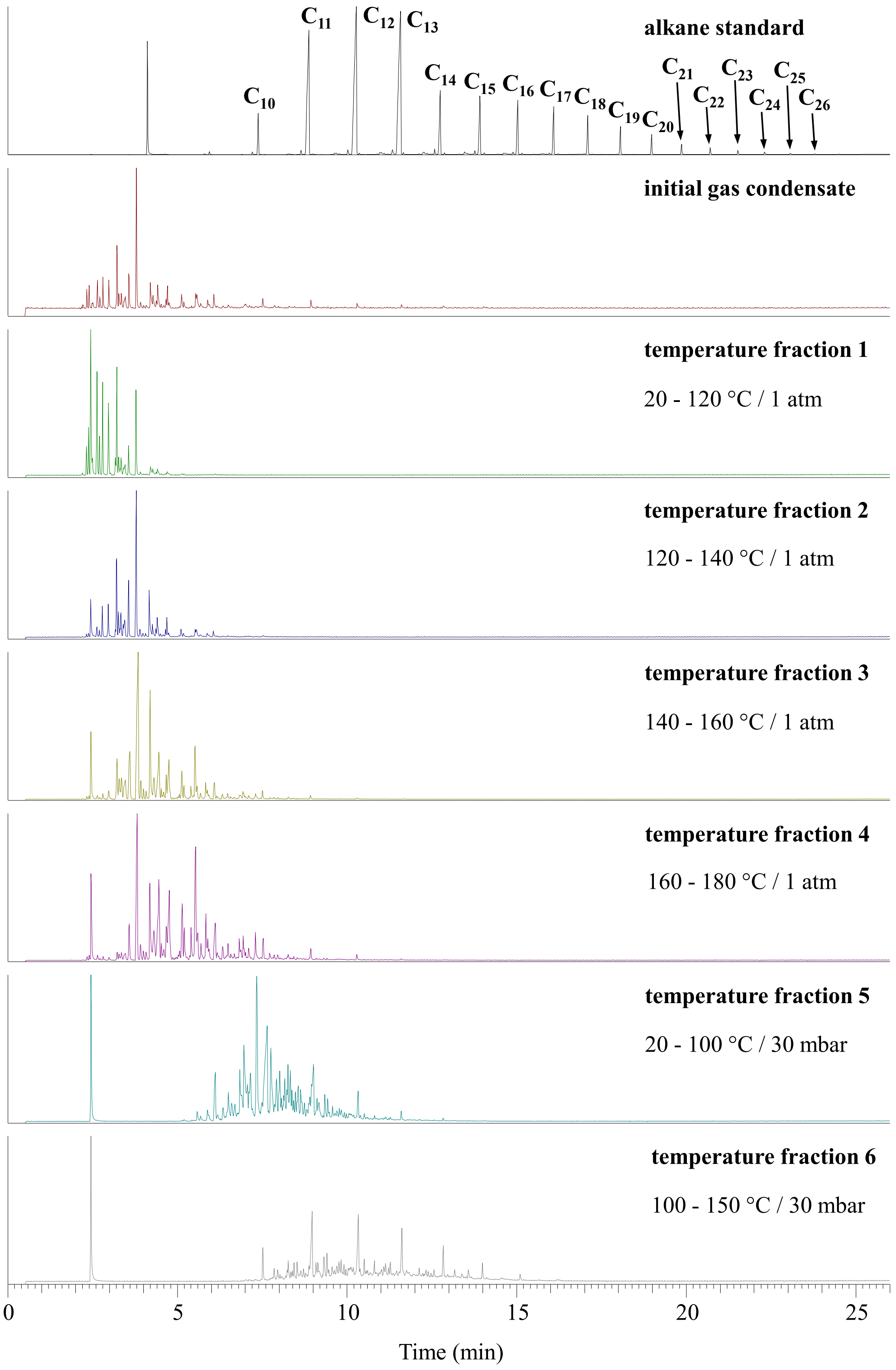

Figure S1. Gas chromatograms (GC-MS) of the initial gas condensate and the respective distillation fractions as well as an alkane standard compound mixture (C_{10}-C_{26}).

Table S1: Summary of compounds in temperature fraction 1 identified by GC-MS and matched by Kovats retention index with GC-FID measurement. The absolute peak area from GC-FID measurement is divided by the carbon count to normalize and calculate the percentage of the certain compound in the mixture. The percentage is calculated in relation to the assigned compounds with GC-MS counting the total absolute peak area of all assigned compounds normalized with the carbon count as 100%. The peak area of the unassigned compounds is excluded from the calculation.

**temperature fraction 1 (20-120°C at 1 atm)**						
**compound**	**Kovats retention index GC-MS**	**Kovats retention index GC-FID**	**C-count**	**absolute peak area [pA*min]**	**peak area per C-atom [pA*min]**	**peak area per C-atom [%] of the total assigned peak area**
hexane isomer	n.b.	n.b.	6	0.84	0.14	13.18
hexane isomer	n.b.	n.b.	6	0.50	0.08	7.75
n-hexane	600.0	600.0	6	1.41	0.23	21.99
C_1 cyclopentane	624.5	624.9	6	0.86	0.14	13.43
benzene	651.7	652.5	6	0.02	0.00	0.34
cyclohexane	658.2	656.7	6	0.92	0.15	14.42
heptane isomer	664.6	663.6	7	0.25	0.04	3.34
heptane isomer	673.4	673.0	7	0.22	0.03	2.99
heptane isomer	688.1	687.4	7	0.12	0.02	1.57
n-heptane	700.0	700.0	7	0.46	0.07	6.13
C_1 cyclohexane	721.4	721.4	7	0.76	0.11	10.24
toluene	759.1	760.4	7	0.14	0.02	1.86
octane isomer	765.8	765.7	8	0.06	0.01	0.70
C_2 cyclohexane	776.5	777.5	8	0.06	0.01	0.72
n-octane	800.0	800.0	8	0.11	0.01	1.34
total peak area [pA*min]	7.719	total assigned peak area [pA*min]	6.726	87.14 % assigned peak area		

Table S2: Summary of compounds in temperature fraction 4 identified by GC-MS and matched by Kovats retention index with GC-FID measurement. The absolute peak area from GC-FID measurement is divided by the carbon count to normalize and calculate the percentage of the certain compound in the mixture. The percentage is calculated in relation to the assigned compounds with GC-MS counting the total absolute peak area of all assigned compounds normalized with the carbon count as 100%. The peak area of the unassigned compounds is excluded from the calculation.

**temperature fraction 4 (160-180°C at 1 atm)**						
**compound**	**Kovats retention index GC-MS**	**Kovats retention index GC-FID**	**C-count**	**absolute peak area [pA*min]**	**C-count corrected peak area [pA*min]**	**peak area per C-atom [%] of the total assigned peak area**
n-heptane	700.0	700.0	7	0.78	0.11	5.48
C_1 cyclohexane	721.9	721.4	7	2.25	0.32	15.71
toluene	755.2	755.1	7	0.61	0.09	4.24
octane isomer	764.3	766.5	8	1.13	0.14	6.94
C_2 cyclohexane	776.4	777.5	8	1.03	0.13	6.27
C_2 cyclohexane	781.8	779.2	8	0.33	0.04	1.99
C_2 cyclohexane	793.3	791.3	8	0.46	0.06	2.83
n-octane	800.0	800.0	8	2.76	0.34	16.88
C_2 benzene	830.8	832.9	8	1.05	0.13	6.40
nonane isomer	850.5	850.4	9	0.25	0.03	1.34
C_2 benzene	860.0	858.8	8	1.35	0.17	8.25
nonane isomer	864.4	867.1	9	0.73	0.08	3.94
C_2 benzene	871.5	873.8	8	0.47	0.06	2.90
C_2 benzene	881.2	880.4	8	0.38	0.05	2.33
n-nonane	900.0	900.0	9	1.69	0.19	9.18
n-decane	1000.0	1000.0	10	0.70	0.07	3.42
n-undecane	1100.0	1100.0	11	0.29	0.03	1.27
n-dodecane	1200.0	1200.0	12	0.11	0.01	0.46
n-tridecane	1300.0	1300.0	13	0.04	0.00	0.15
total peak area [pA*min]	24.69	total assigned peak area [pA*min]	16.38	66.37 % assigned peak area		

Table S3: Summary of compounds in temperature fraction 5 identified by GC-MS and matched by Kovats retention index with GC-FID measurement. The absolute peak area from GC-FID measurement is divided by the carbon count to normalize and calculate the percentage of the certain compound in the mixture. The percentage is calculated in relation to the assigned compounds with GC-MS counting the total absolute peak area of all assigned compounds normalized with the carbon count as 100%. The peak area of the unassigned compounds is excluded from the calculation.

**temperature fraction 5 (20-100°C at 30 mbar)**						
**compound**	**Kovats retention index GC-MS**	**Kovats retention index GC-FID**	**C-count**	**absolute peak area [pA*min]**	**C-count corrected peak area [pA*min]**	**peak area per C-atom [%] of the total assigned peak area**
n-nonane	900.0	900.0	9	0.34	0.04	2.36
C_3 benzene	949.8	950.2	9	0.11	0.01	0.77
C_3 benzene	958.3	957.6	9	0.47	0.05	3.24
C_3 benzene	967.2	967.2	9	0.57	0.06	3.95
decane isomer	970.3	970.7	9	0.11	0.01	0.75
C_3 benzene	981.5	980.6	9	0.36	0.04	2.49
n-decane	1000.0	1000.0	10	4.13	0.41	25.90
C_4 benzene	1008.7	1008.9	10	0.14	0.01	0.89
C_4 benzene	1011.9	1012.2	10	0.12	0.01	0.73
C_4 cyclohexane	1022.0	1022.8	10	0.33	0.03	2.05
C_4 cyclohexane	1028.9	1026.1	10	0.68	0.07	4.25
C_4 cyclohexane	1032.7	1031.2	10	0.70	0.07	4.38
C_4 benzene	1037.9	1038.2	10	0.24	0.02	1.50
C_4 benzene	1040.2	1039.5	10	0.19	0.02	1.17
C_4 benzene	1044.7	1043.9	10	0.11	0.01	0.70
C_4 benzene	1046.9	1047.0	10	0.27	0.03	1.66
decalin	1052.0	1051.3	10	0.40	0.04	2.50
C_4 benzene	1055.0	1055.0	10	0.25	0.02	1.54
C_4 benzene	1065.8	1064.0	10	0.45	0.04	2.79
C_4 benzene	1068.7	1066.9	10	0.58	0.06	3.65
C_4 benzene	1074.4	1073.4	10	0.52	0.05	3.25
C_5 benzene	1093.2	1094.5	11	0.25	0.02	1.44
n-undecane	1100.0	1100.0	11	2.70	0.25	15.40
C_1 decalin	1108.9	1106.2	11	0.26	0.02	1.48

C_5 benzene	1112.9	1111.0	11	0.14	0.01	0.78
C_1 decalin	1126.3	1123.9	11	0.24	0.02	1.38
C_5 benzene	1159.2	1158.9	11	0.17	0.02	0.99
n-dodecane	1200.0	1200.0	12	1.06	0.09	5.56
n-tridecane	1300.0	1300.0	13	0.39	0.03	1.87
n-tetradecane	1400.0	1400.0	14	0.13	0.01	0.58
total peak area [pA*min]	28.54	total assigned peak area [pA*min]	16.36		57.33 % assigned peak area	

Table S4: Summary of compounds in temperature fraction 6 identified by GC-MS and matched by Kovats retention index with GC-FID measurement. The absolute peak area from GC-FID measurement is divided by the carbon count to normalize and calculate the percentage of the certain compound in the mixture. The percentage is calculated in relation to the assigned compounds with GC-MS counting the total absolute peak area of all assigned compounds normalized with the carbon count as 100%. The peak area of the unassigned compounds is excluded from the calculation.

**temperature fraction 6 (100-150°C at 30 mbar)**						
**compound**	**Kovats retention index GC-MS**	**Kovats retention index GC-FID**	**C-count**	**absolute peak area [pA*min]**	**C-count corrected peak area [pA*min]**	**peak area per C-atom [%] of the total assigned peak area**
n-decane	1000.0	1000.0	10	0.73	0.07	7.01
undecane isomer	1025.1	1026.3	11	0.17	0.02	1.51
C_4 cyclohexane	1031.5	1032.5	10	0.17	0.02	1.60
C_4 benzene	1045.4	1042.1	10	0.12	0.01	1.11
decalin	1047.6	1048.1	10	0.08	0.01	0.78
undecane isomer	1052.0	1054.0	11	0.17	0.02	1.50
C_4 benzene	1066.5	1067.0	10	0.29	0.03	2.82
C_5 benzene	1074.4	1073.4	10	0.24	0.02	2.28
C_5 cyclohexane	1084.2	1084.7	11	0.12	0.01	1.02
C_5 benzene	1091.1	1090.3	11	0.08	0.01	0.67
n-undecane	1100.0	1100.0	11	2.05	0.19	17.94
C_1 decalin	1111.7	1109.7	11	0.19	0.02	1.67
C_1 decalin	1126.9	1126.5	11	0.23	0.02	1.98

C_5 cyclohexane	1133.7	1135.1	11	0.27	0.02	2.39
C_5 benzene	1137.4	1140.0	11	0.09	0.01	0.74
C_5 benzene	1145.6	1147.8	11	0.12	0.01	1.07
C_5 benzene	1159.4	1158.8	11	0.16	0.01	1.37
C_6 cyclohexane	1164.4	1162.2	12	0.22	0.02	1.78
naphthalin	1184.17	1187.00	10	0.17	0.02	1.62
n-dodecane	1200.0	1200.0	12	1.77	0.15	14.22
C_6 cyclohexane	1214.0	1214.7	12	0.65	0.05	5.21
C_6 cyclohexane	1238.1	1239.5	12	0.15	0.01	1.23
C_6 cyclohexane	1274.6	1277.1	12	0.27	0.02	2.13
n-tridecane	1300.0	1300.0	13	1.10	0.08	8.16
C_1 naphthalin	1311.1	1313.8	11	0.07	0.01	0.59
C_7 cyclohexane	1343.0	1343.2	13	0.12	0.01	0.87
tetradecane isomer	1378.7	1380.7	14	0.12	0.01	0.84
n-tetradecane	1400.0	1400.0	14	0.56	0.04	3.82
C_2 decalin	1429.3	1429.6	15	0.64	0.04	4.11
pentadecane isomer	1464.8	1466.6	15	0.13	0.01	0.83
n-pentadecane	1500.0	1500.0	15	0.25	0.02	1.57
n-hexadecane	1600.0	1600.0	16	0.90	0.06	5.41
n-heptadecane	1700.0	1700.0	17	0.03	0.00	0.16
total peak area [pA*min]	17.81	total assigned peak area [pA*min]	12.23		68.67 % assigned peak area	

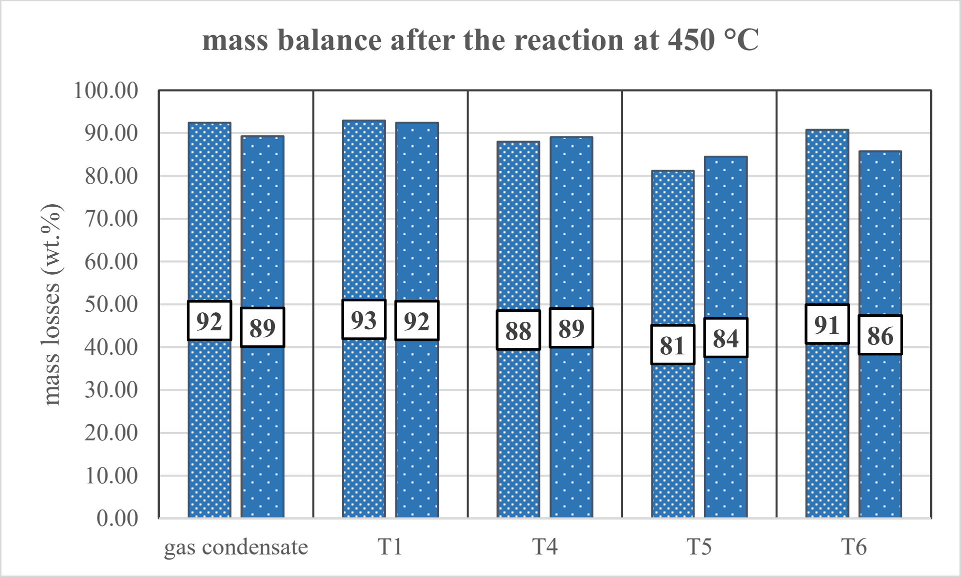

Figure S2: Mass balances of the reaction of the gas condensate, T1, T4, T5 and T6 at 450°C. Both bars represent the same sample in repeat determination and the numbers at the center of the bars represents the mass loss in weight percent. The sample was weighed in the quartz crucibles before and after the reaction. Additionally, the residual condensate in the metal autoclave was weighed and added to the total weight of the product.

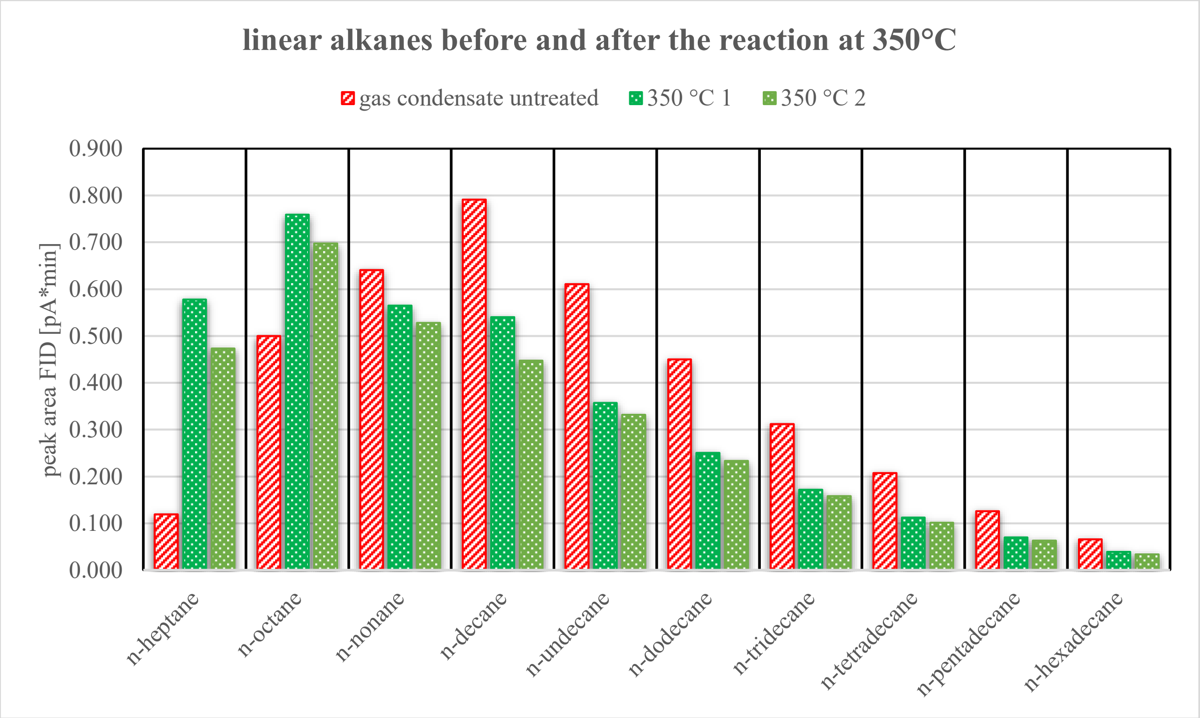

Figure S3: Peak areas from GC-FID measurement of linear hydrocarbons in the initial gas condensate (red bar) and after the reaction at 350 °C in repeat determination (green bars). The compounds were identified with GC-MS and matched with an alkane standard compound standard mixture.

Fragmentation spectra

The fragmentation energy is converted from the dimensionless normalized collision energy (NCE) into the actual collision energy [eV] by equation 1. The charge factor in this case is one since only singly charged ions were dectected.

$$collision\ energy\ [eV] = \frac{NCE\ \cdot molar\ mass}{500\ \cdot charge\ factor}$$

The shown fragment structures are possible outcomes of the fragmentation and may differ from the actual structure. Alkylated structures may have methyl, ethyl or propyl chains depending of the number of carbon atoms in the side chain with unknown positioning at the certain molecules since only MS^2 fragmentation was performed.

The fragmentation results indicate high energy dissociation channels as C_2H_2 or H_2 losses in some cases. As an example naphthalene shows a C_2H_2 loss yielding a C_8H_6 fragment which most likely either is a benzocyclobutadiene or a phenylacetylene following theoretical calculations for the lowest energy dissociation channel (S4).[1] The naphthalene derivatives $C_{12}H_{12}$ and $C_{13}H_{14}$ also indicate C_2H_2 losses, however, only of the beforehand formed tropylium ions (S5-6). The formation of tropylium ions is a typical fragmentation reaction of alkylated polycyclic aromatic hydrocarbons (PAHs).[2] The consecutive loss of C_2H_2 was not reported in the literature so far. The same behavior can be observed for the phenanthrene derivatives $C_{15}H_{12}$ and $C_{16}H_{14}$ as well as for the pyrene or fluoranthene derivatives $C_{17}H_{12}$ and $C_{18}H_{14}$ (S7-8 and S10-11). However, the pyrene or fluoranthene derivative $C_{19}H_{16}$ indicate no C_2H_2 losses instead it only shows the formation of tropylium ions (S12). The only difference between $C_{19}H_{16}$ and $C_{17}H_{12}$ as well as $C_{18}H_{14}$ is the collision energy which is 16 eV or rather 17 eV lower for $C_{19}H_{16}$. The energy difference seems to be sufficient to induce C_2H_2 losses. Another high energy fragmentation pathway is the loss of H_2 which theoretically is estimated to become a relevant dissociation channel at internal PAH temperatures about 2200 K.[3] This would suggest PAH ions in the keV region may undergo such fragmentation reactions. Interestingly, this fragmentation pattern can be observed for the phenanthrene derivatives $C_{15}H_{12}$ and $C_{16}H_{14}$ as well as for the structure of either pyrene or fluoranthene (S7-9).

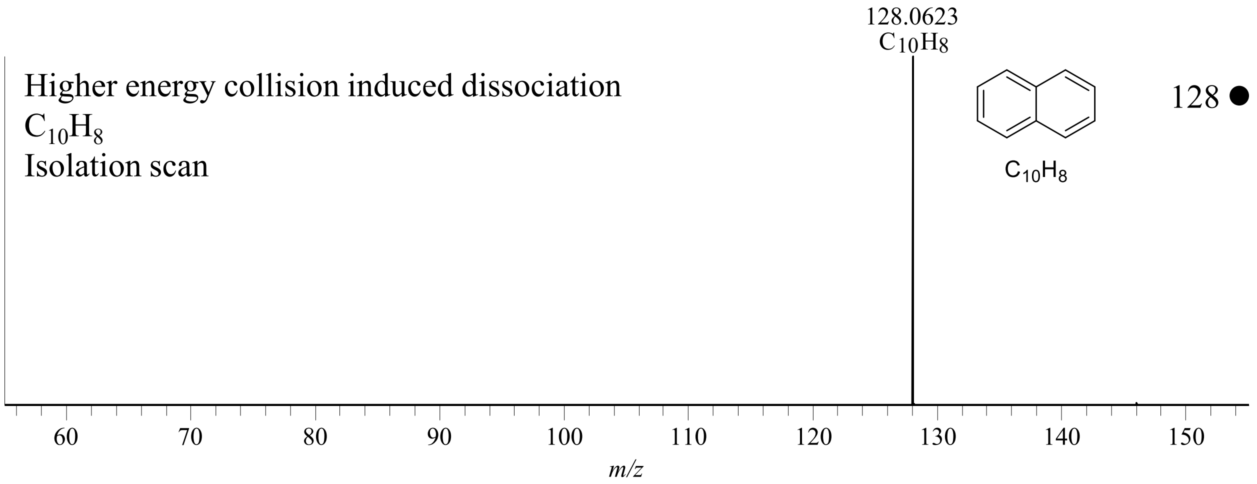

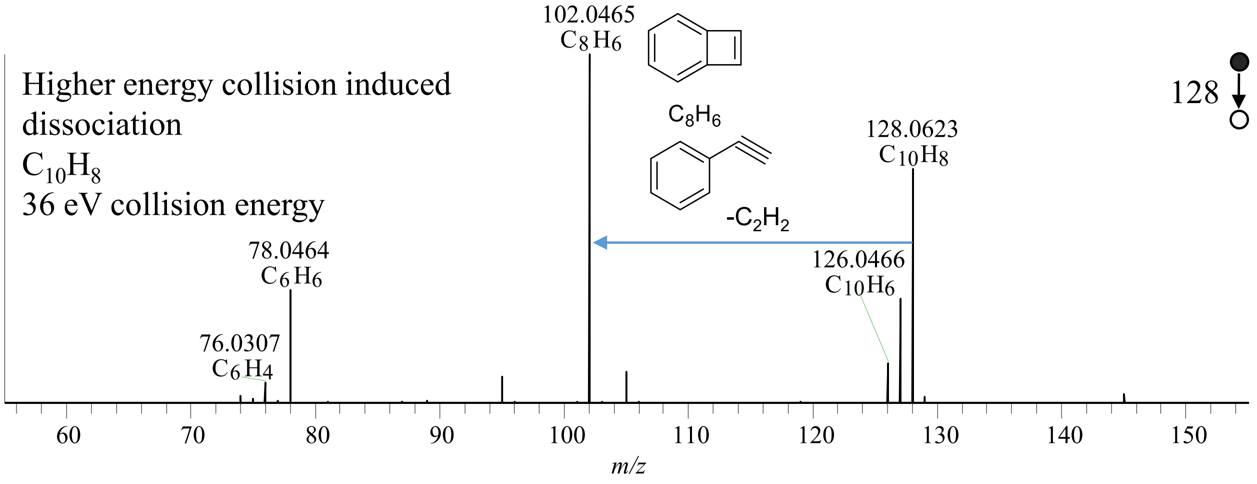

Figure S4: Fragmentation spectra of $C_{10}H_8$ (lower spectrum) using higher energy collision induced dissociation (HCD). The upper spectrum shows the isolation scan at a mass of 128.06 Da with an isolation window of 0.4 Da.

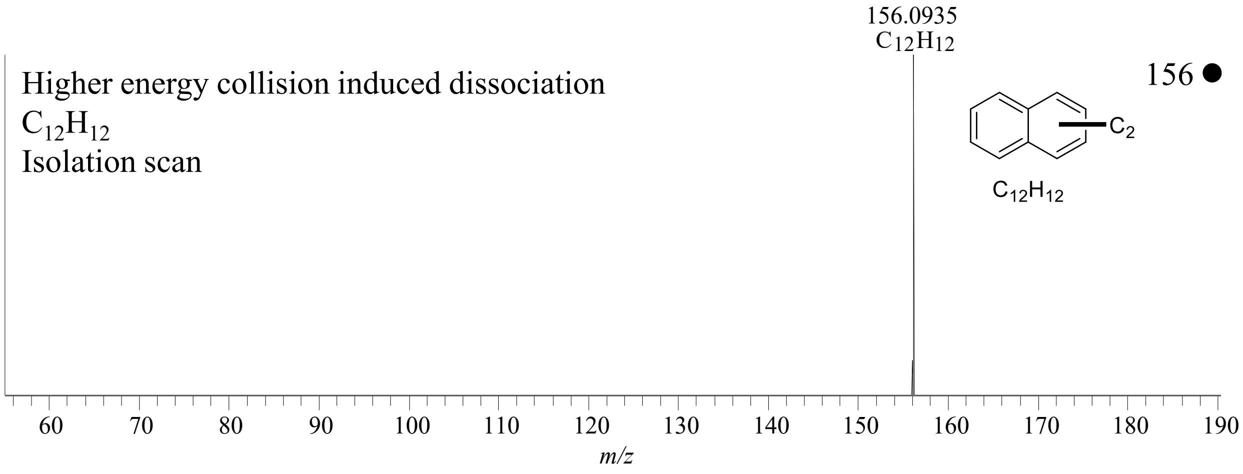

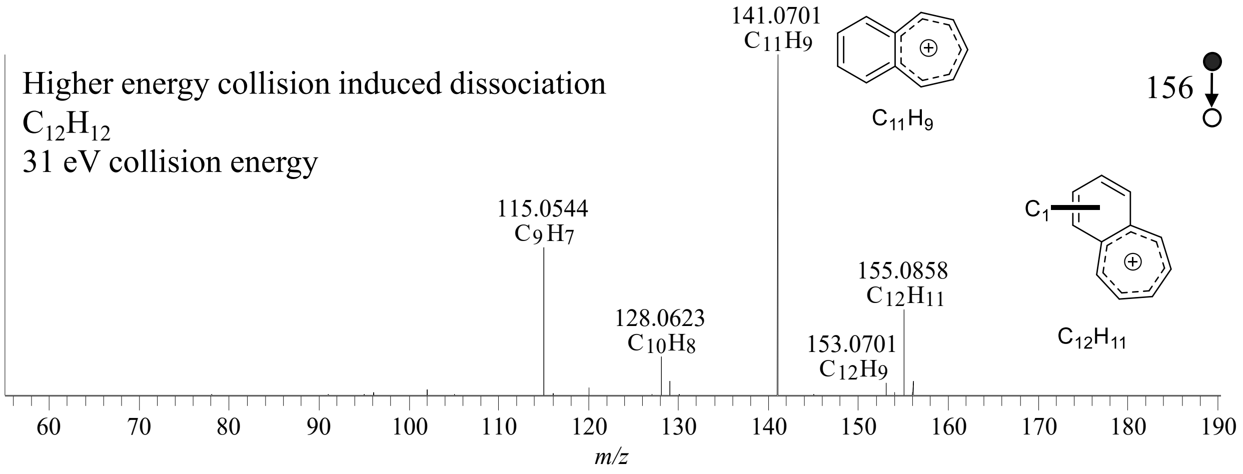

Figure S5: Fragmentation spectra of $C_{12}H_{12}$ (lower spectrum) using higher energy collision induced dissociation (HCD). The upper spectrum shows the isolation scan at a mass of 156.09 Da with an isolation window of 0.4 Da.

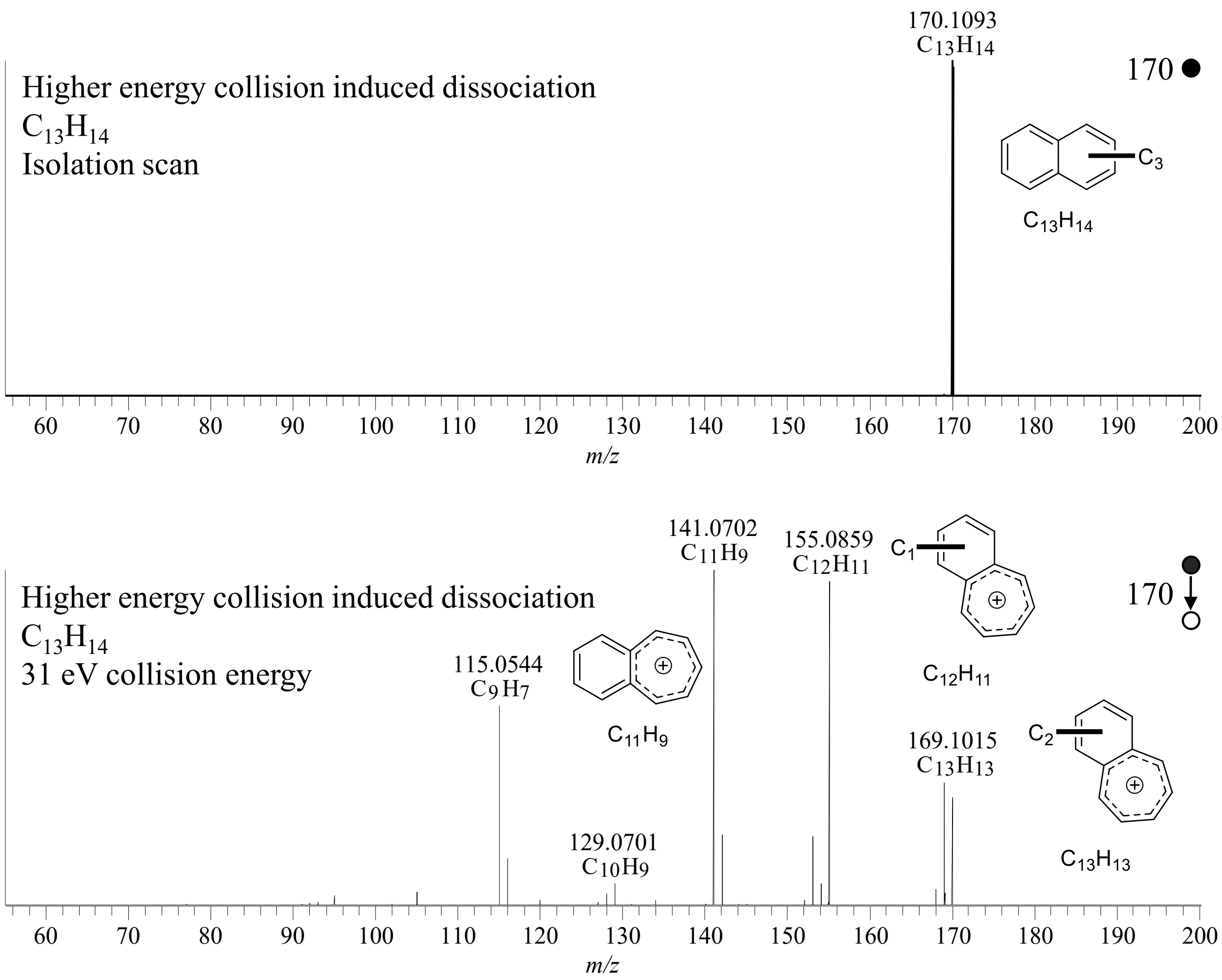

Figure S6: Fragmentation spectra of $C_{13}H_{14}$ (lower spectrum) using higher energy collision induced dissociation (HCD). The upper spectrum shows the isolation scan at a mass of 170.10 Da with an isolation window of 0.4 Da.

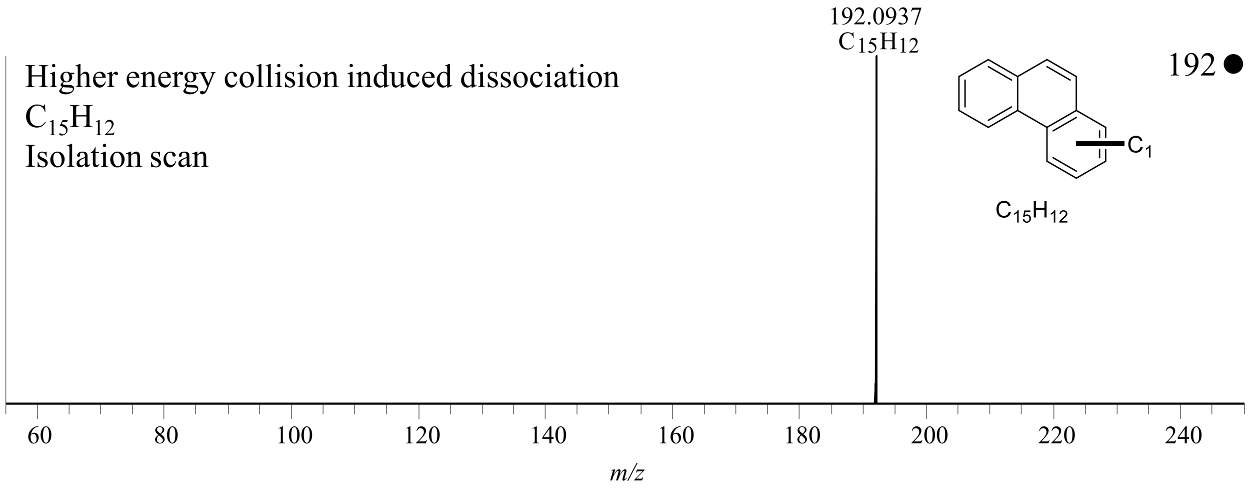

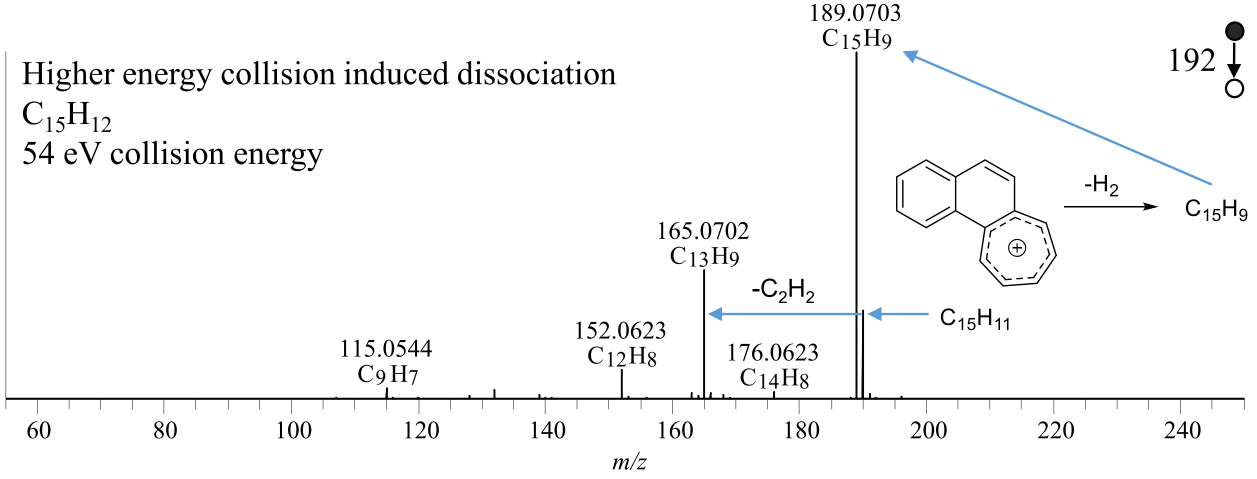

Figure S7: Fragmentation spectra of $C_{15}H_{12}$ (lower spectrum) using higher energy collision induced dissociation (HCD). The upper spectrum shows the isolation scan at a mass of 192.09 Da with an isolation window of 0.4 Da.

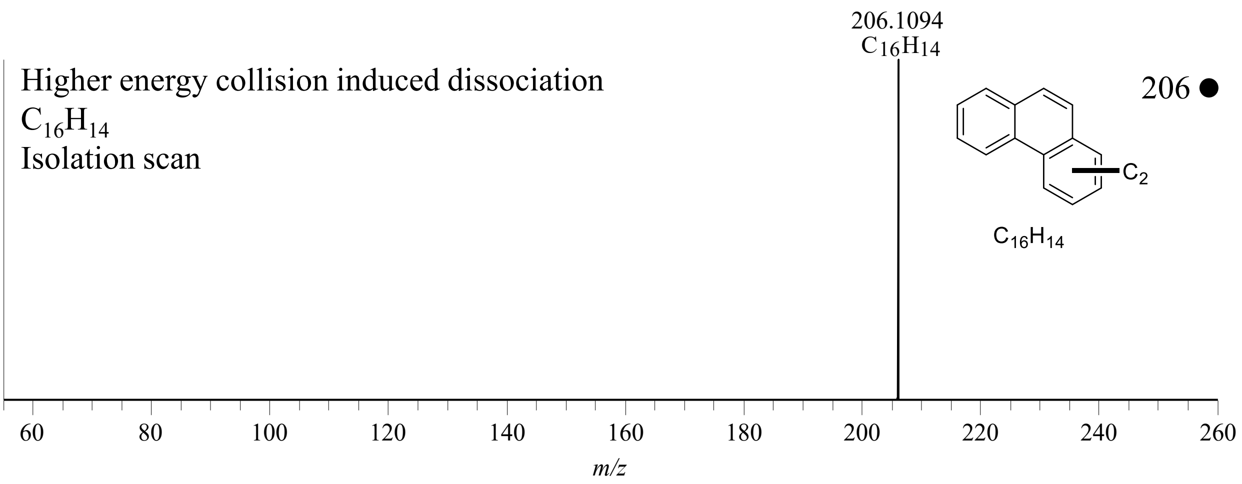

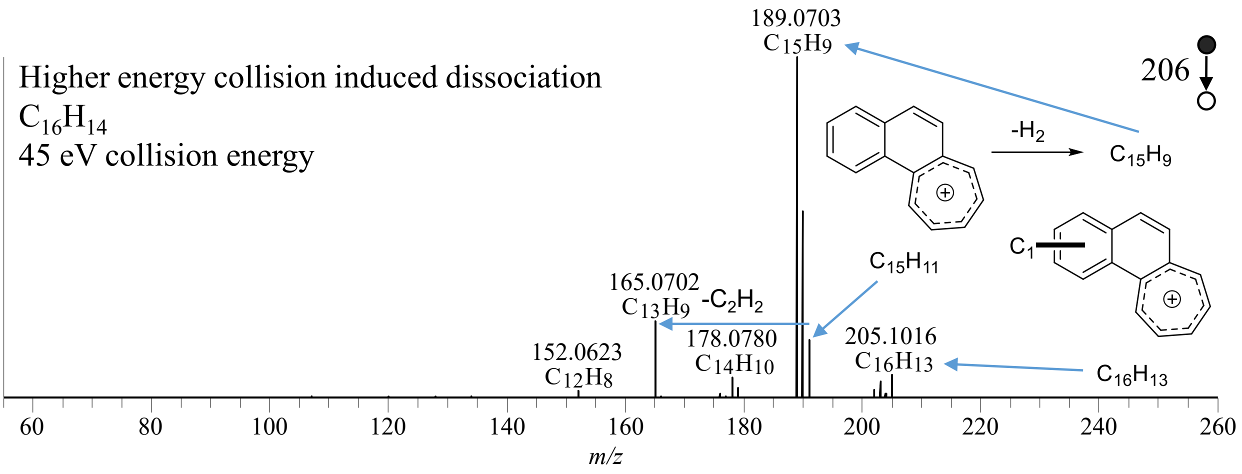

Figure S8: Fragmentation spectra of $C_{16}H_{14}$ (lower spectrum) using higher energy collision induced dissociation (HCD). The upper spectrum shows the isolation scan at a mass of 206.10 Da with an isolation window of 0.4 Da.

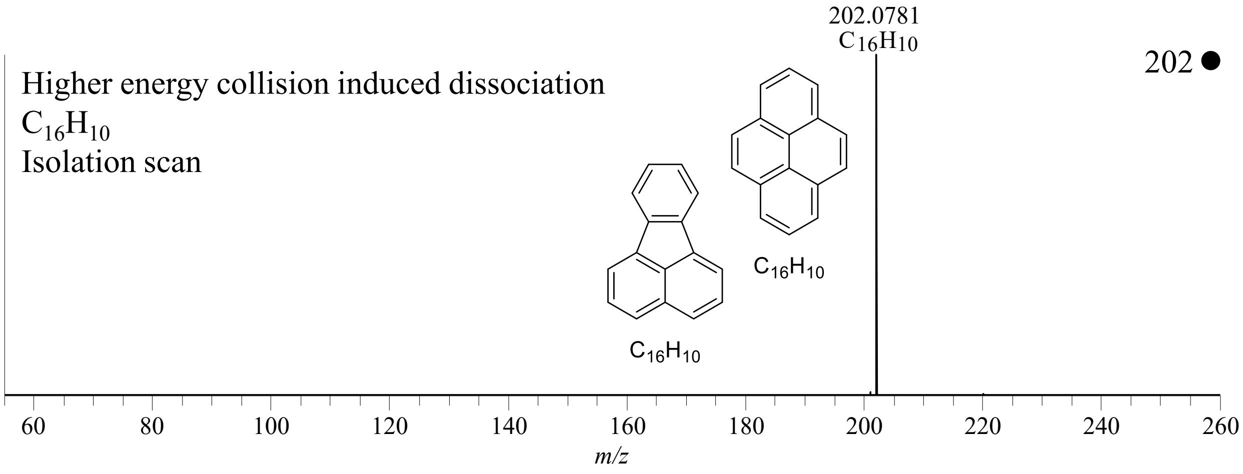

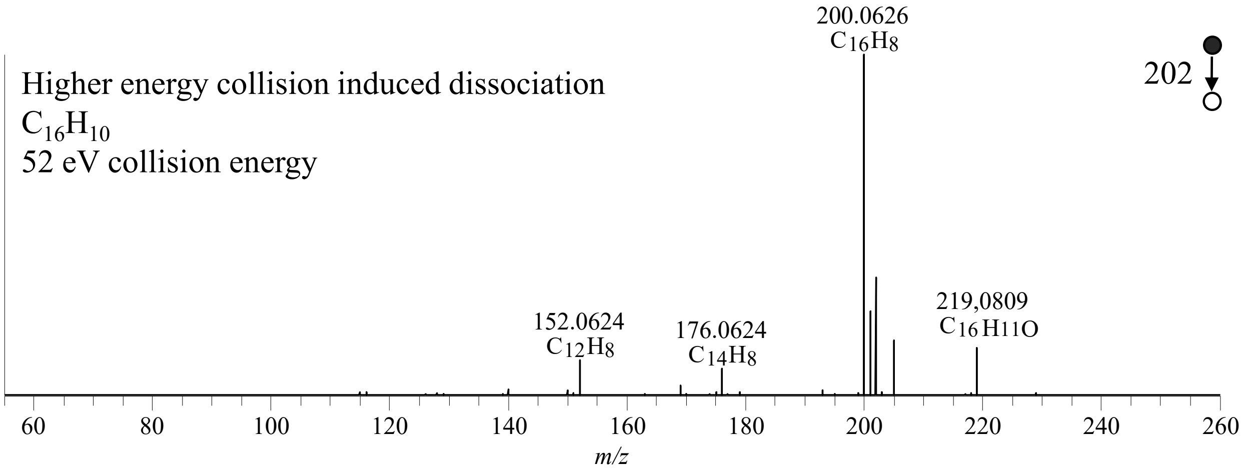

Figure S9: Fragmentation spectra of $C_{16}H_{10}$ (lower spectrum) using higher energy collision induced dissociation (HCD). The upper spectrum shows the isolation scan at a mass of 202.07 Da with an isolation window of 0.4 Da.

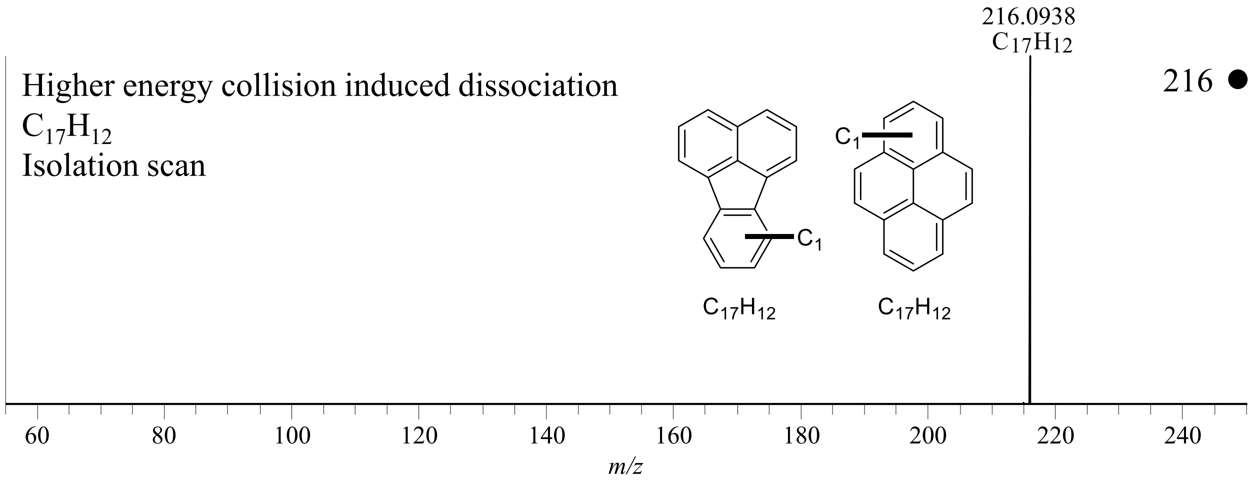

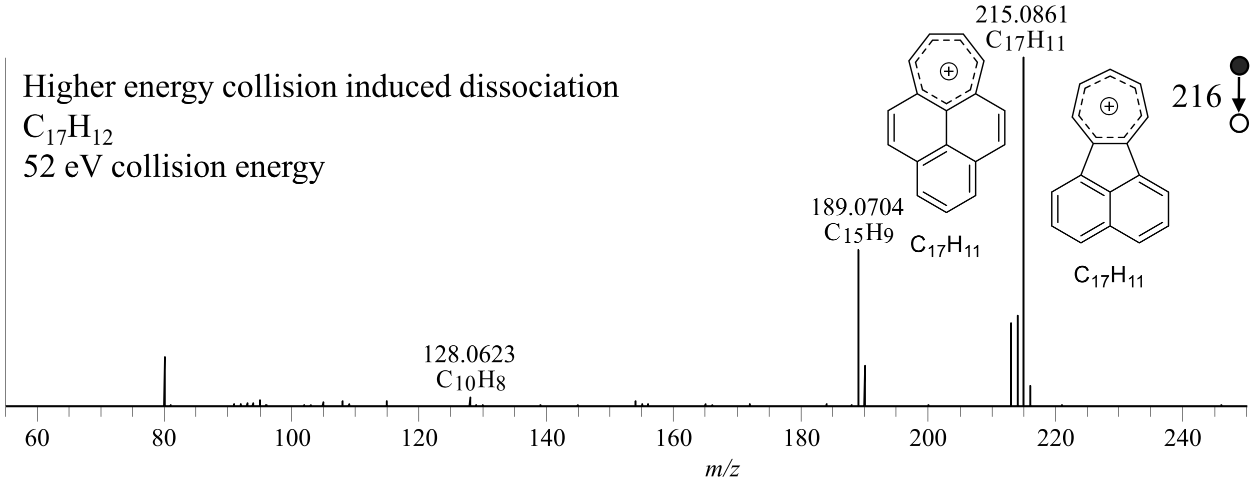

Figure S10: Fragmentation spectra of C17H12 (lower spectrum) using higher energy collision induced dissociation (HCD). The upper spectrum shows the isolation scan at a mass of 216.09 Da with an isolation window of 0.4 Da.

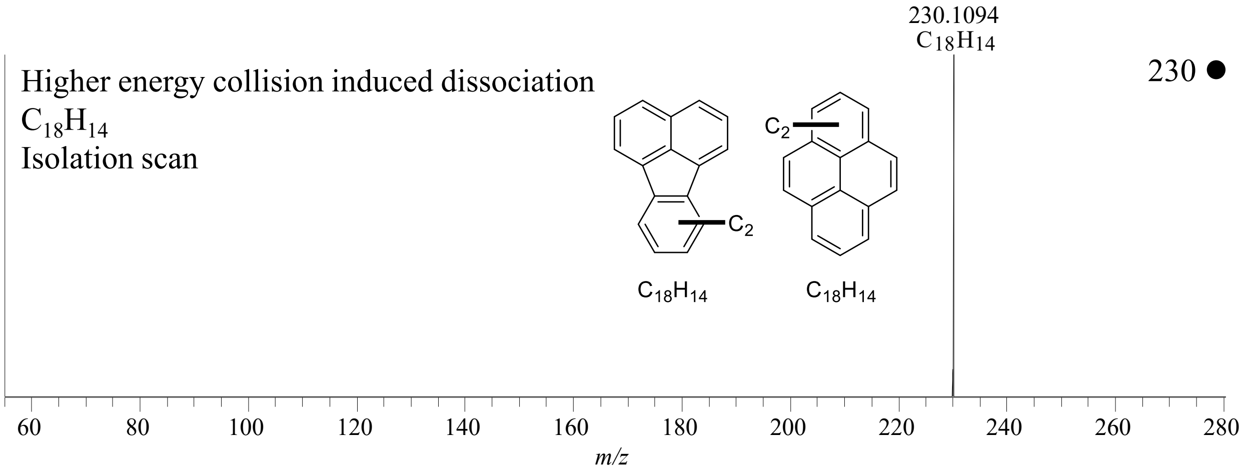

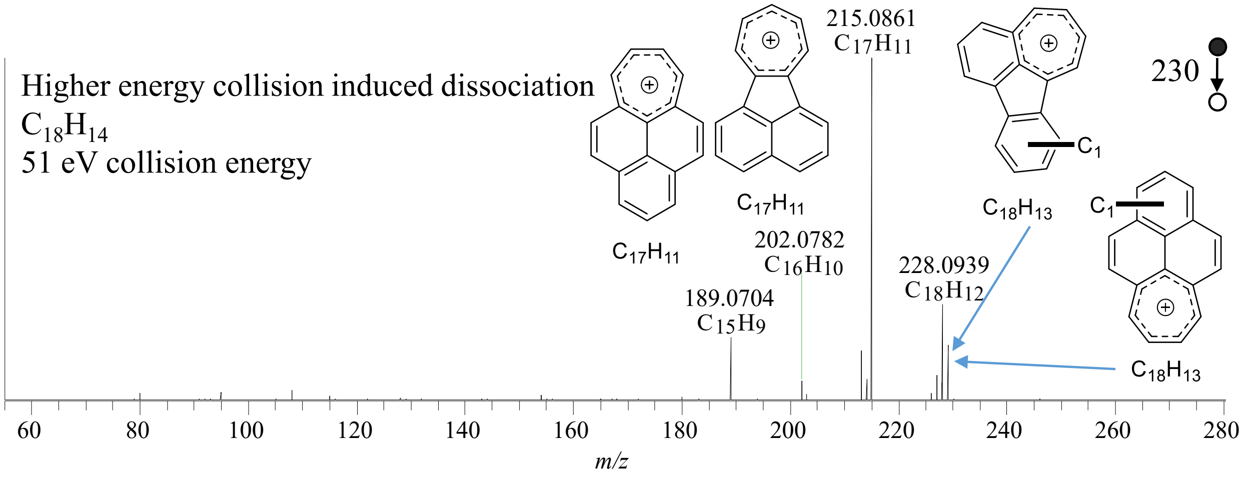

Figure S11: Fragmentation spectra of $C_{18}H_{14}$ (lower spectrum) using higher energy collision induced dissociation (HCD). The upper spectrum shows the isolation scan at a mass of 230.10 Da with an isolation window of 0.4 Da.

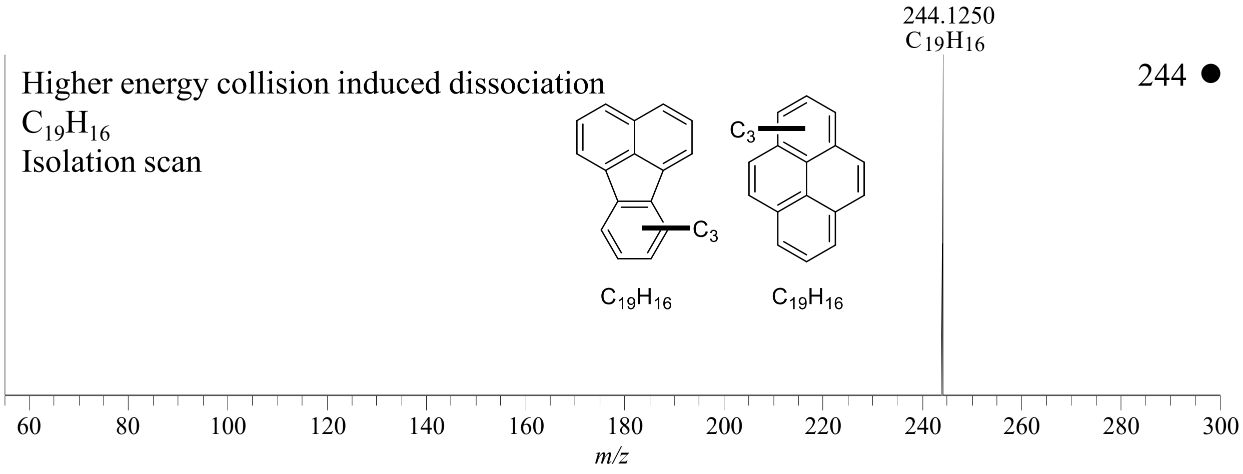

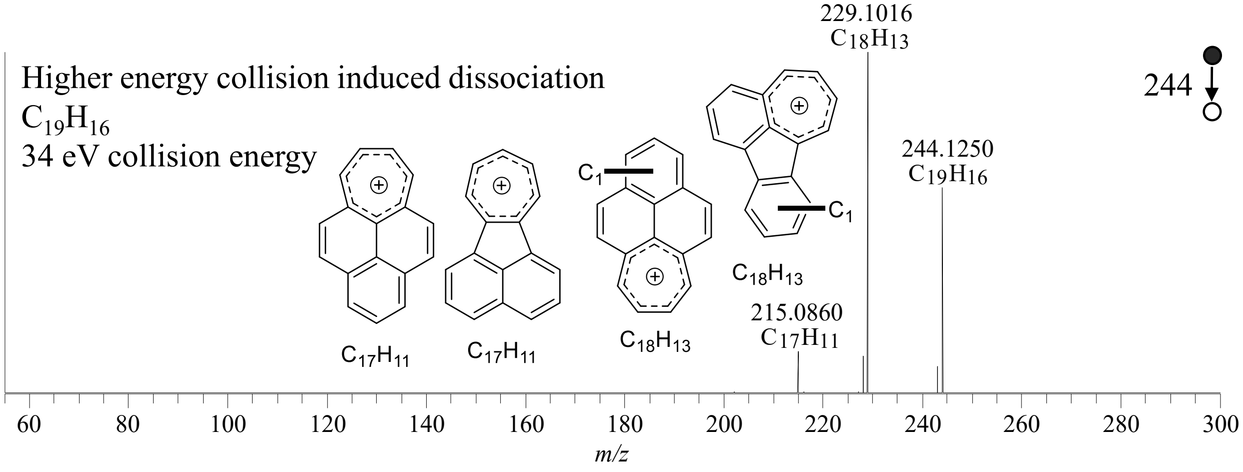

Figure S12: Fragmentation spectra of $C_{19}H_{16}$ (lower spectrum) using higher energy collision induced dissociation (HCD). The upper spectrum shows the isolation scan at a mass of 244.12 Da with an isolation window of 0.4 Da.

1. Holm, A. I. S.; Johansson, H. A. B., et al., Dissociation and multiple ionization energies for five polycyclic aromatic hydrocarbon molecules. *J. Chem. Phys.* **2011,** *134* (4), 10.1063/1.3541252.

2. Vetere, A.; Alachraf, M. W., et al., Studying the fragmentation mechanism of selected components present in crude oil by collision-induced dissociation mass spectrometry. *Rapid Commun. Mass Spectrom.* **2018,** *32* (24), 2141-2151, 10.1002/rcm.8280.

3. Chen, T.; Gatchell, M., et al., Formation of H2 from internally heated polycyclic aromatic hydrocarbons: Excitation energy dependence. *J. Chem. Phys.* **2015,** *142* (14), 10.1063/1.4917021.
